# Seasonal and Sexual Variations in Corticosterone and Total Triiodothyronine: A Pilot Study in Mediterranean Tortoises (*Testudo hermanni*)

**DOI:** 10.3390/ani14192810

**Published:** 2024-09-29

**Authors:** Sergi Olvera-Maneu, Xavier Navarro, Paula Serres-Corral, Annaïs Carbajal, Albert Martínez-Silvestre, Manel López-Béjar

**Affiliations:** 1 Department of Veterinary Medicine, University of Nicosia School of Veterinary Medicine, 2414 Nicosia, Cyprus; 2 Centro Veterinario Los Sauces, 28010 Madrid, Spain; xavinme@gmail.com; 3 Department of Animal Health and Anatomy, Universitat Autònoma de Barcelona, 08193 Bellaterra, Spain; paula.serres@uab.cat (P.S.-C.); anais.carbajal@uab.cat (A.C.); manel.lopez.bejar@uab.cat (M.L.-B.); 4 Catalonian Reptiles and Amphibians Rescue Center (CRARC), 08783 Masquefa, Spain; crarc@amasquefa.com

**Keywords:** adrenal, thyroid, stress, reptile, chelonian, annual variation, conservation, homeostasis

## Abstract

**Simple Summary:**

The Hermann’s tortoise (*Testudo hermanni*), or Mediterranean tortoise, is one of the two native species of terrestrial tortoises that inhabit the Iberian Peninsula. This is a vulnerable species protected by legislation and subjected to conservation and captive breeding programs. The present study is the first to simultaneously evaluate the four-season variations in corticosterone and total triiodothyronine in semi-captive adults and juvenile individuals of Western Hermann’s tortoises (*T. hermanni hermanni*). The current investigation revealed that corticosterone concentrations changed seasonally depending on the sex, whereas total triiodothyronine changed seasonally in both sexes. Juvenile individuals did not show seasonal variations in any of the evaluated hormones. Overall, this study adds evidence and provides novel information about the seasonal variations in corticosterone and total triiodothyronine in adult and juvenile individuals of this protected species.

**Abstract:**

The Mediterranean tortoise *Testudo hermanni* inhabits different regions bordering the northwestern Mediterranean. This species is vulnerable, protected by legislation, and involved in various breeding and reintroduction programs. Wild populations face numerous environmental and anthropogenic stressors that can potentially interfere with their conservation. While seasonal changes in stress-response biomarkers, such as glucocorticoids and thyroid hormones, have been widely studised in mammals and birds, there is a paucity of research in reptile species. Therefore, the present study aimed to evaluate the seasonal fluctuations in corticosterone and total triiodothyronine levels in adult and juvenile Hermann’s tortoises (*Testudo hermanni)* as a measure of the physiological stress response. Blood samples were collected seasonally (winter, spring, summer, and autumn) and posteriorly analyzed by using a specific and validated enzyme immunoassay for both hormones, respectively. The results showed that corticosterone levels varied seasonally and differed between sexes, whereas total triiodothyronine levels changed seasonally but did not differ between sexes. Notably, juveniles exhibited no seasonal changes in either corticosterone or total triiodothyronine levels. Additionally, no correlation between blood extraction duration and hormonal concentrations was observed. This study is pioneering in its comprehensive evaluation of corticosterone and total triiodothyronine changes across all four seasons, including winter, and its focus on juvenile Hermann’s tortoises.

## 1. Introduction

The Hermann’s tortoise (*Testudo hermanni*) is one of the two native species of terrestrial tortoises inhabiting the Iberian Peninsula. These tortoises are found along the northern Mediterranean coast, although natural populations are scarce, largely influenced by human-induced activities and interactions. Currently, two distinct subspecies of Mediterranean tortoises have been identified: *Testudo hermanni hermanni* and *Testudo hermanni boettgeri*. The first subspecies inhabits the countries and islands bordering the northwest Mediterranean (Spain, France, and Italy), whereas the latter is present in the eastern Mediterranean. Fossil and genetic evidence suggest that current populations from the western Mediterranean Islands were likely reintroduced through human transportation from Sicily several thousand years ago after having become extinct in these regions [1,2,3]. It must be noted that different cases of hybridization between these subspecies have been detected in continental France, Spain, and Italy, whereas, in the Mediterranean islands, it appears to be absent [1,4,5,6] These cases are believed to be caused by human intervention rather than natural causes. Nowadays, *Testudo hermanni* has been included in the appendix II by the Convention on International Trade in Endangered Species of Wild Fauna and Flora (CITES). They are protected by legislation and involved in various conservation and captive breeding programs to recover natural populations across several European countries [5,7].

Hormonal changes play a crucial role in regulating a wide range of physiological and behavioral processes, including the stress response, metabolism, and reproduction, in many animal species [8,9]. More specifically, corticosterone (CORT), the final effector released into the bloodstream after the activation of the hypothalamic–pituitary–adrenal (HPA) axis, has long been considered the primary hormone to assess the stress response in reptiles [8,10,11]. The stress response is also mediated by the activation of the sympathetic nervous system, with the final release of catecholamines, preparing the body for fight or flight. The combined effect of these two hormonal groups, catecholamines and glucocorticoids, increases available energy and oxygen consumption, decreases blood flow to areas not critical for movement, and suppresses digestion, growth, immune, reproductive, and pain functions, among others [12]. In contrast, triiodothyronine, now onwards referred to as tT3, is a thyroid hormone mainly involved in metabolic regulation [13]. Understanding the relationship between CORT and T3 in Mediterranean tortoises is essential for comprehending how these reptiles respond to different seasonal stressors and regulate their metabolism. Even though these hormones have distinct roles, they often interact in complex ways [14,15]. Animals must adapt their physiological and behavioral responses to seasonal environmental changes to regulate their energy balance, among other processes. These adaptations are often driven by hormones that exhibit seasonal variation patterns, enabling animals to anticipate and respond to forthcoming environmental changes like hibernation, reproduction, or migration [11,16]. While seasonal changes in glucocorticoids, like CORT, and thyroid hormones, like tT3, have been extensively studied in mammals and birds, research in reptile species remains limited [11,16,17].

As previously stated, variations in baseline CORT levels in reptiles are relatively sparse compared to mammals and birds, yet findings indicate diverse patterns across species [11,17]. Romero [17] described that approximately 70% of the examined reptile species exhibit seasonal variations with varying patterns. In brief, three hypotheses have been proposed for these seasonal fluctuations: (1) “energy mobilization,” where glucocorticoids would be higher when the reptiles need more energy, such as during the breeding season; (2) “behavioral adaptation,” where higher glucocorticoid levels would enable reptiles to perform certain behaviors at specific periods; and (3) “preparation”, where glucocorticoid levels would help the individuals prepare for future stressful situations. Understanding how baseline CORT levels vary seasonally in Hermann’s tortoises could help researchers and conservationists to better interpret the physiological stress responses of these reptiles and implement effective conservation strategies [9]. On the other hand, thyroid hormones are involved in processes such as ecdysis, growth, development, nutrient assimilation, tail regeneration, activity, or metabolic rate regulation [13,18,19]. They also play significant roles in reproductive behavior [19], gonadal maturation [20], and the cardiovascular system [21]. Various factors, including intrinsic elements such as age, sex, and season [18,22], illness, stress [21], or external factors like temperature [13,23], photoperiod, or reproductive events, can potentially influence thyroid hormone fluctuations [18,22,24,25]. Studies on hibernating reptiles similar to *Testudo* spp. suggest that decreased thyroid hormone levels during hibernation correlate with a lowered metabolic rate, while higher levels may aid in ending hibernation and resuming activity [21].

Reptiles exhibit distinct life stages from juveniles to adults, each characterized by unique physiological demands and developmental requirements [26]. Age-related differences in hormonal levels are thus an important aspect of reptilian endocrinology [27,28]. Juvenile and adult tortoises may exhibit different hormonal profiles due to their varying physiological needs and developmental stages [26,28,29]. Juveniles are often in a phase of rapid growth and development, requiring different metabolic and hormonal demands in comparison to adults, who focus more effort on reproduction and maintaining homeostasis [30]. However, there is a lack of evidence on how these hormonal levels vary between age groups in Hermann’s tortoises, particularly in relation to seasonal changes.

The present study examines seasonal and sexual fluctuations in CORT and tT3 levels in adult and juvenile Hermann’s tortoises (*Testudo hermanni)* as a measure of the physiological stress response. The authors hypothesized that there would be significant seasonal fluctuations in CORT and tT3 levels in both adult and juvenile studied individuals corresponding to changes in environmental conditions, sexual differences, and physiological needs throughout the year.

## 2. Materials and Methods

### 2.1. Individuals, Location, and Management Conditions

A total of 22 healthy individuals of Mediterranean tortoises *Testudo hermanni hermanni* (9 females, 7 males, and 6 juveniles) were included in this study. Table 1 presents the weight measurements of both adult and juvenile tortoises across each season, providing a comparative overview of their development. Juvenile individuals were not sexed since secondary sexual characteristics were not yet developed. The animals were housed in a wildlife rehabilitation center, CRARC (Catalonian Reptiles and Amphibians Rescue Center), located in Masquefa (Catalonia, Spain). All animals belonged to a stable group of the center and were not moved from their normal location during the studied period. The adult male and female tortoises were housed in the same enclosure of 50 m^2^ with a lake, natural substrate, and native vegetation mimicking the wild Mediterranean habitats. The juvenile tortoises were housed in a different enclosure of 1 m^2^ with natural substrate, vegetation, and available water. Both enclosures had several places and vegetation where the tortoises could hide and shelter and were outdoors with natural exposure to ambient fluctuations in temperature, daylight, humidity, and rainfall, among others. The animals received vegetal feeding once per day during the non-hibernating seasons (spring, summer, and autumn). Finally, all individuals were identified with a permanent paint specifically designed for this purpose in order to facilitate their identification throughout the duration of this study.

### 2.2. Blood Sampling and Ethics

The present study did not require approval from the ethics committee, as the samples were reused from a veterinary diagnostic procedure conducted periodically at the recovery center (CRARC). This procedure was part of the routine care of the animals and did not involve any additional risk or manipulation beyond the standard practices for the subjects involved. A physical examination was performed by the responsible veterinarian on all animals to certify their general health status before each sampling. All animals were healthy during the studied period. The sampling procedure was performed once per season, and all samples were collected within a specific time frame (from 9 a.m. to 1 p.m.) to minimize the impact of potential circadian rhythm influence and ensure consistency in the collected data. Winter samples were collected on 27 January, spring samples were collected on 3 May, summer samples were collected on 10 September, and finally, autumn samples were collected on 17 November 2021. For the winter samples, the animals were individually captured by hand and removed from the shelter where they were hibernating, and blood samples were then taken. After finishing the procedure, the animals were immediately relocated to the same place. Regarding the other sampling periods (spring, summer, and autumn), the tortoises were located around the enclosure, individually captured by hand, and sampled immediately. The adult blood samples were collected from the dorsal coccygeal vein, the subcaparacial vein, or the occipital venous sinus using a 2 mL heparinized sterile syringe with a 23G needle. For the juveniles, the blood samples were collected from the subcarapacial sinus using a 1 mL heparinized sterile syringe with a 25G needle. The blood samples were placed into lithium heparin blood collection tubes (Eurotubo^®^ Deltalab S.L, Barcelona, Spain). The duration of the blood extraction procedure for the adults and juveniles is presented in Table 2. The sample tubes were maintained refrigerated on ice until the arrival at the laboratory and centrifuged at 6000 rpm for 15 min at room temperature. Plasma was stored in microtubes and frozen at −20 °C until further analysis.

### 2.3. Hormone Quantification and Assay Validation

CORT concentration levels were determined by using the corticosterone enzyme immunoassay detection kit (Neogen Corporation*^®^*, Ayr, UK). This test provides a quantitative analysis of CORT levels based on competition between the enzyme conjugate and the CORT present in the sample for a limited number of binding sites on an antibody-coated plate. The tT3 concentration levels were determined by using the total triiodothyronine enzyme immunoassay detection kit (IBL International GMBH*^®^*, Ham, Germany). The assay validations were performed by using a constituted pool created with 50 μL from all the plasma samples included in this study. The precision of the CORT and tT3 tests was assessed by calculating the coefficient of variation (CV) from all duplicated samples. For CORT, the specificity of the test was assessed by calculating the linearity of the dilution using 1:1, 1:2, 1:8, and 1:10 dilutions of the pool with the enzyme immunoassay buffer. The accuracy was assessed through the spike-and-recovery test, calculated by adding, to 100, 75, and 25 μL of the pool, volumes of 25, 75, and 100 μL of three standard CORT concentrations provided by the kit (0.5, 1, and 2 ng/mL). For tT3, the same procedure as CORT was followed. The linearity of the dilution was assessed using 1:1, 1:2, 1:4, 1:6, and 1:8 dilutions of the pool with the kit buffer. The spike-and-recovery test was calculated by adding, to 100, 75, and 25 μL of the pool, volumes of 25, 75, and 100 μL of three standard tT3 concentrations provided by the kit (1, 2.5, and 5 ng/mL). Finally, the sensitivity of the test was given by the smallest amount of CORT and tT3 detected by the assays.

### 2.4. Statistical Analysis

Data were analyzed using R software (Version 4.3.0, R Development Core Team) and graphically represented with GraphPad Prism (Version 9.5.1; Graph Pad Software Inc., Boston, MA, USA). The statistically significant level was established at *p* < 0.05 for all performed tests. For the data obtained in the dilution test and the spike-and-recovery test of the EIA biochemical validations, a Pearson’s Product Moment correlation was applied between the expected and obtained values for plasma CORT and tT3. Data normal distribution was evaluated using the D’Agostino skewness test and the Bonett–Seier test for kurtosis. The Levene test was performed to evaluate the equality of variances. Data were log transformed to achieve normality. A Linear Mixed Model (lme4 package) [31] was performed to evaluate the seasonal variations in plasma CORT levels of adults, setting as fixed factors the sex (male or female), the season (winter, spring, summer, and autumn), and the interaction between both factors. The individuals were set as a random factor. Akaike’s information criteria corrected for small sample size (AICc) were used to select the most parsimonious model based upon the ΔAICc and AICc weights. The same function and criteria were used to study the seasonal variations in plasma tT3 levels. For CORT, post hoc tests were performed using the emmeans R package [32] to evaluate the seasonal differences between and within sexes. To evaluate seasonal differences in tT3 concentrations, a Tukey post hoc test was used. The seasonal variations in CORT and tT3 levels in juvenile individuals were evaluated by a linear mixed effect model, setting the season as a fixed factor and the individual as a random factor for both hormones, respectively. The relation between the extraction time and the duration with the hormonal concentrations was assessed by a Pearson product moment correlation. Data are presented as the mean ± SEM unless otherwise stated.

## 3. Results

### 3.1. Assay Validations

For CORT, the coefficient of variation was 15.50%. In the linearity of dilution, the obtained CORT concentrations correlated with the expected CORT values (r = 0.98; *p* < 0.05). In the spike-and-recovery test, the hormone standards spiked with the samples pool presented a mean recovery percentage of 118.01 ± 21.55% (mean ± SD). For tT3, the coefficient of variation was 5.40%. In the linearity of dilution, the obtained T3 concentrations correlated with the expected tT3 values (r = 0.99; *p* < 0.05). In the spike-and-recovery test, the hormone standards spiked with the samples pool presented a mean recovery percentage of 118.82 ± 16.24% (mean ± SD). The sensitivity of the test was 0.37 and 0.44 nmol/L for CORT and tT3, respectively. Overall, the results of the biochemical validation of the enzyme immunoassays showed reliable results and demonstrated the assays’ precision, specificity, accuracy, and sensitivity in measuring *Testudo hermanni* plasma CORT and tT3.

### 3.2. CORT and tT3 Seasonal Variations

**Figure 1:**
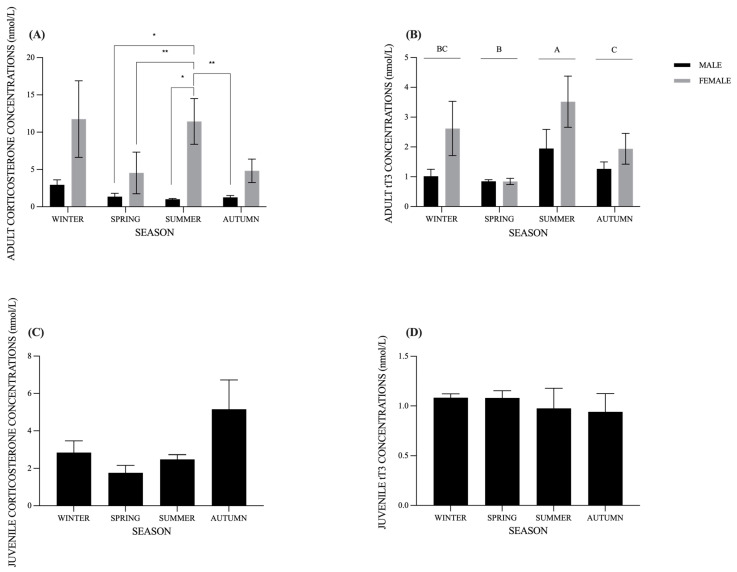
(**A**) Seasonal variations in CORT concentrations (nmol/L) in male (*n* = 7) and female (*n* = 9) adults of *T. hermanni*. Data are presented as mean ± SEM. Asterisks represent significant differences (* for *p* < 0.05; ** for *p* < 0.01). (**B**) Seasonal variations in tT3 concentrations (nmol/L) in male and female adults of *T. hermanni*. Different uppercase letters represent significant seasonal differences (*p* < 0.05). (**C**) Seasonal CORT concentrations (nmol/L) in juvenile individuals (*n* = 6) of *T. hermanni*. (**D**) Seasonal tT3 concentrations (nmol/L) in juvenile individuals (*n* = 6) of *T. hermanni*.

The corticosterone and tT3 concentrations for the adults in winter, spring, summer, and autumn are presented in Figure 1. According to our model selection, the best candidate model explaining the observed corticosterone variability included the interaction between season and sex (df = 10; AICc = 66.70; ΔAICc = 0.00; weight = 0.441), indicating that corticosterone concentrations in the evaluated individuals were significantly influenced by sex (*p* < 0.01) and the interaction between season and sex (*p* < 0.05). Post hoc comparisons of the interaction between season and sex revealed significant differences that are presented in Figure 1A.

For tT3, according to our model selection, the best candidate model explaining the observed corticosterone variability included the additive effects of season and sex (df = 7; AICc = 27.30; ΔAICc = 0.00; weight = 0.553). The plasma tT3 concentrations were significantly affected by the season (*p* < 0.001) but, contrary to corticosterone, not for sex (*p* > 0.05). The seasonal differences in plasma tT3 concentrations are presented in Figure 1B. The juveniles did not present significant differences between seasons neither for corticosterone nor tT3 (Figure 1C,D). Finally, no correlation was detected between the duration of blood extraction and the concentrations of either hormone, CORT or tT3 (*p* > 0.05).

## 4. Discussion

The present study is the first to simultaneously evaluate the four-season variations in CORT and tT3 concentrations in semi-captive adult and juvenile individuals of Western Hermann’s tortoises (*T. hermanni*). Seasonal CORT concentrations exhibited sex-dependent fluctuations, while tT3 concentrations exhibited similar seasonal changes in both sexes. The juveniles did not show seasonal variations in either of the evaluated hormones. Overall, this study provides novel insights into the seasonal hormonal fluctuations of CORT and tT3 in both adult and juvenile individuals of *T. hermanni*, with implications for reptile medicine and conservation programs. However, some limitations must be considered. First, one sample per season was collected, and a small number of animals were included (*n* = 22), which may affect the generalizability of the results. On the other hand, this research was conducted in a single geographic region (Catalonia) and a controlled environment (CRARC), which may not fully reflect natural conditions as well as the adaptation level of individuals from other regions. Additionally, the sex of the juvenile individuals was not able to be determined since they had not yet developed secondary sexual characteristics [33]. Future research could benefit from larger sample sizes, diverse geographic regions, natural settings, and sex determination of the juvenile individuals.

Previous research has reported variable results regarding seasonal CORT changes in tortoises of different species. While Ott et al. [34] reported no significant seasonal variations in CORT during the studied periods, while other studies have observed such seasonal fluctuations [35,36,37,38]. Another factor worth considering is the sex of the individuals, as numerous studies have revealed an influence on the CORT levels [35,36,37,38]. Our results showed that CORT concentrations changed seasonally in females but not in males, having higher CORT concentrations in summer compared to spring. Following this line, females presented higher CORT concentrations in summer compared with the observed results in males during spring, summer, and autumn. Thus, females had higher corticosterone concentrations in the active season that could be addressed to prepare them for potential future adverse or stressful periods [17]. These findings are not consistent with those observed by Sibeaux et al. [36], who reported increased CORT values in spring compared to summer in females of Mediterranean tortoises as well as higher CORT values in males compared to females during the studied period. The significance of the sex and season interaction could reflect a seasonal difference of reproductive effort between males and females noted by the seasonal differences observed between the two sexes [35,36,37,38]. Temperature is thought to also influence CORT concentrations in reptiles. However, different results have been observed. For instance, lower body temperatures resulted in lower baseline and stress-induced CORT levels in eastern fence lizards *(Sceloporus undulatus)* [39], while in Children’s pythons *(Antaresia childreni)*, cold temperatures led to higher baseline and stress-induced levels, potentially aiding in maintaining an alert state [40]. Overall, these findings suggest that CORT responses in tortoises may vary depending on species and environmental conditions. To date, the present study is the first to evaluate CORT concentrations during the hibernation period in Mediterranean tortoises. Contrary to expected, no significant seasonal variation for males and females was detected when comparing hibernation and post-hibernation periods (winter vs. other periods). However, the results obtained in winter were characterized by a high variability between individuals, suggesting a different secretion pattern between them. Further research is needed to clarify the role of corticosterone in the hibernation metabolism of Mediterranean tortoises.

Corticosterone, as an indicator of the stress response, is subjected to potential increases when individuals are captured/manipulated for blood sample collection [11]. However, in the present study, no correlation between manipulation time and CORT concentrations was observed. In line with our results, Kahn et al. [41] did not detect plasma CORT variations in Gopher tortoises (*Gopherus polyphemus*) after blood sampling. On the other hand, Drake et al. [38]demonstrated, after an ACTH test with captive desert tortoises (*Gopherus agassizii*), that ACTH-injected animals did not show an increase in plasma CORT until approximately 20 min post-injection, and there was no increase in plasma CORT levels in saline-injected animals within 60 min. However, the duration associated with an acute stress reaction may be influenced by environmental and animal-based conditions [38]. In the present study, the manipulation time ranged from 59.7 ± 5.56 s (41–83; min–max) in juveniles in summer to 262 ± 95.8 s (106–797; min–max) in males in autumn. Notably, our findings suggested no immediate effect of manipulation on CORT concentrations, indicating that the obtained measures represented the baseline CORT concentrations for the Hermann’s tortoise studied population.

In reptiles, thyroid hormones influence biological processes, like reproduction, growth development, or nutrient assimilation, and activity [13,18,19]. In general, reptiles have plasma concentrations of thyroid hormones that are far lower than those of mammals [21,42,43]. Different studies have reported that tT3 plasma concentrations in chelonians were under the detection limit [21,29], contrary to what was observed in the present study, where tT3 concentrations were successfully measured for all the evaluated individuals, both adults and juveniles. Additionally, the assay validation demonstrated the assay’s precision, specificity, accuracy, and sensitivity in measuring plasma tT3 in Hermann’s tortoises. Our findings indicated that plasma tT3 concentrations were significantly influenced by the season in adults, showing the highest tT3 concentrations in summer compared to the other seasons. In addition, the obtained results described, for the first time, the tT3 concentrations during the hibernation period of this species. Seasonal fluctuations of thyroid hormones are common in reptiles and are thought to be essential for the proper functioning of their thyroid gland. Consequently, in many reptile species including tortoises, thyroid activity peaks during the warmer months and declines during the colder months, suggesting a relationship between temperature, food availability, and hormone concentrations [14,18,24,29,44]. The fluctuations in tT3 concentrations observed in the present study would reflect the species’ adaptive response to environmental changes. In addition, the present results indicate that these fluctuations remained consistent between sexes, suggesting a shared physiological response to seasonal variations rather than sex-specific hormonal regulation, contrary to what was observed for CORT concentrations.

Age-related differences in hormonal levels are an important aspect of reptilian endocrinology [21,27,28]. However, the information regarding seasonal hormonal fluctuations in juvenile reptiles, and more specifically in tortoises, is scarce, probably because of the difficulty in obtaining blood samples [11]. The present study is the first to evaluate potential seasonal fluctuations of CORT and tT3 in juvenile individuals of Hermann’s tortoises, and contrary to expectations, no significant variations were observed for either CORT or tT3. This result contrasts with our findings in adult individuals of the same species. Previous studies have reported that CORT levels in juvenile reptiles vary depending on the species and environmental factors. Research on juvenile American alligators showed that CORT levels fluctuated seasonally [27]. Similarly, significant seasonal variations in CORT concentrations were observed in wild juvenile female tuatara but not in wild juvenile males [45]. On the other hand, Currylow et al. [28] demonstrated, in juvenile wild radiated tortoises, a marked seasonal pattern, obtaining CORT concentrations for juveniles twice as high as those in adults. The reasons for the absence of significant seasonal variations in juvenile individuals observed in the present study remain unclear, and further research is needed to clarify this observation. Overall, these findings highlight the complexity of seasonal hormonal variations in juvenile reptiles, potentially influenced by factors such as captivity, environmental stress, and species-specific responses, therefore, emphasizing the importance of understanding these dynamics for conservation and management strategies.

## 5. Conclusions

This study is pioneering in its evaluation of the seasonal dynamics of corticosterone (CORT) and total triiodothyronine (tT3) across all four seasons, including the hibernation period, in both adult and juvenile individuals of Hermann’s tortoises. The results of the present study described a sex-dependent seasonal fluctuation in CORT concentrations among adults, while tT3 levels exhibited consistent seasonal changes across both sexes. Notably, juveniles exhibited no seasonal changes in either CORT or tT3 levels. In addition, hormonal concentrations were not influenced by the duration of sampling. These findings highlight the complexity of hormonal regulation in response to seasonal changes and underscore the necessity for further research into the environmental and physiological factors influencing these hormonal dynamics in reptiles. Understanding these factors is crucial for developing effective conservation and management strategies for this vulnerable species.

## Figures and Tables

**Table 1:** Weight across seasons (g) for females, males, and juveniles of the studied individuals. Data are presented as mean ± SEM along with the range (minimum to maximum).

	Winter	Spring	Summer	Autumn
Female (*n* = 9)	907 ± 92.5 (493–1415)	919 ± 101 (500–1408)	958 ± 93.4 (518–1432)	926 ± 103 (478–1379)
Male (*n* = 7)	557 ± 67.1 (339–830)	622 ± 80.1 (357–851)	606 ± 49.6 (378–807)	588 ± 52.9 (351–791)
Juveniles (*n* = 6)	104 ± 4.8 (84–117)	113 ± 7.7 (91–135)	107 ± 7.7 (83–138)	97.8 ± 8.3 (75–127)

**Table 2:** Blood extraction duration (seconds) across seasons for females, males, and juveniles of the studied individuals. Data are presented as mean ± SEM along with the range (minimum to maximum).

	Winter	Spring	Summer	Autumn
Female (*n* = 9)	191 ± 18.1 (123–273)	161.1 ± 31.7 (52–302)	165.2 ± 63.5 (52–656)	242.6 ± 98 (81–909)
Male (*n* = 7)	139.3 ± 14.3 (74–184)	82.1 ± 7.7 (52–117)	103.3 ± 28.9 (28–308)	262 ± 95.8 (106–797)
Juveniles (*n* = 6)	176.5 ± 20 (122–147)	96.5 ± 23.3 (63–211)	59.7 ± 5.6 (41–83)	144 ± 44.1 (88–320)

## Data Availability

The data presented in this paper have not been published or stored elsewhere but are available upon request from S.O.-M.
